# Warfarin Reversal With Four-Factor Prothrombin Complex Concentrate for Left Ventricular Assist Device Conversion Surgery Monitored by TEG6s: A Case Report

**DOI:** 10.7759/cureus.27745

**Published:** 2022-08-07

**Authors:** Hisakatsu Ito, Akiyo Kameyama, Masaaki Kawakami, Shigeki Yokoyama, Mitsuaki Yamazaki

**Affiliations:** 1 Anesthesiology, Toyama University Hospital, Toyama, JPN; 2 Department of Anesthesiology, University of Toyama, Toyama, JPN; 3 Department of Thoracic and Cardiovascular Surgery, University of Toyama, Toyama, JPN; 4 Anesthesiology, Toyama Nishi General Hospital, Toyama, JPN

**Keywords:** point-of-care monitoring, left ventricular assist devices, warfarin reversal, thromboelastography, four-factor prothrombin complex concentrate

## Abstract

Left ventricular assist devices (LVADs) require careful therapeutic anticoagulation with warfarin to prevent pump thrombosis. However, the best method to reverse warfarin for elective LVAD conversion surgery with massive bleeding remains unclear. We report the case of a 39-year-old Japanese man who was administered a four-factor prothrombin complex concentrate (4F-PCC) as warfarin reversal when he underwent conversion surgery from paracorporeal LVAD to implantable LVAD. 4F-PCC with co-administration of vitamin K reduced the international normalized ratio and R time in TEG6s (Haemonetics Corporation, USA) immediately. The effect was prolonged, and good hemostasis was achieved. 4F-PCC with vitamin K provided good hemostasis in our patient; therefore, 4F-PCC could be a useful tool for elective LVAD conversion surgery with expected massive bleeding and requiring immediate warfarin reversal.

## Introduction

A left ventricular assist device (LVAD) requires careful therapeutic anticoagulation with warfarin to prevent pump thrombosis. Although prompt warfarin reversal is often required for patients with LVAD to undergo invasive procedures, including LVAD conversion surgery, the best method remains unclear. Four-factor prothrombin complex concentrate (4F-PCC) includes a vitamin K-dependent coagulation factor, and the effects appear immediately [1]. Although the current indication for 4F-PCC use is only for urgent surgery [1,2], it can be reasonable for patients who underwent elective LVAD conversion. On the other hand, thromboembolic complications following 4F-PCC administration can be a concern [3,4]; therefore, point-of-care (POC) monitoring is recommended to monitor the coagulation status frequently [5]. Nevertheless, there is limited clinical data regarding the efficacy of POC monitoring with 4F-PCC use in elective surgery with LVAD.

## Case presentation

We report the case of a 39-year-old Japanese man (173 cm, 50.0 kg) who was administered 4F-PCC as warfarin reversal to undergo conversion surgery from paracorporeal LVAD to implantable LVAD. He was diagnosed with dilated cardiomyopathy when he was 29 years old. Because an infection caused acute exacerbation of congestive heart failure (ejection fraction was 20%), we implanted a paracorporeal LVAD as a bridge to recovery. It improved multiple organ failure but did not recover cardiac function. Therefore, we scheduled an LVAD conversion surgery. Because a white thrombus already existed in the pump circuit, the patient had been taking warfarin 5 mg/day, aspirin 100 mg/day, and heparin 10,000 U/day. The prothrombin time-international normalized ratio (PT-INR) was 2.64, and the activated partial thromboplastin time (APTT) was 62.9.

Massive bleeding was expected during this surgery due to a history of multiple sternotomies. Our multidisciplinary heart team, including cardiac surgeons, cardiologists, neurosurgeons, anesthesiologists, and perfusionist for mechanical circulatory support, decided to continue warfarin until the morning of surgery and to administer 4F-PCC with vitamin K. We injected 1,250 U of 4F-PCC based on PT-INR and body weight immediately before surgery, followed by 20 mg of vitamin K at two hours before the surgery. Effective hemostasis was obtained, and the calculated blood loss was 220 mL due to adhesiotomy and driveline creation.

**Figure 1:**
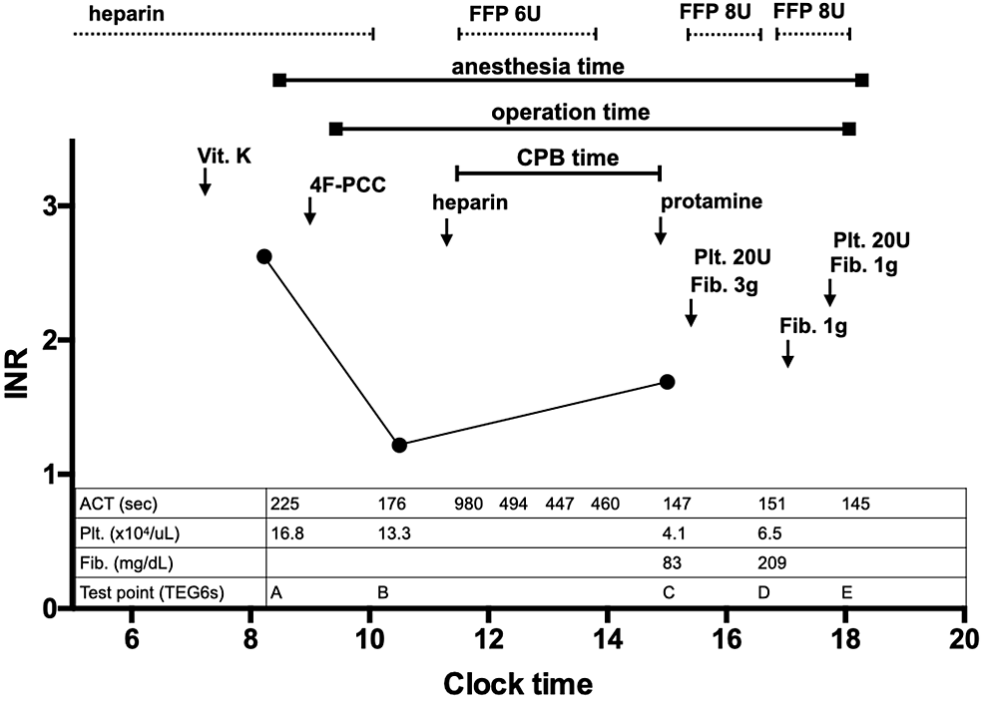
Test point of TEG and time changes in PT-INR during the operation. Blood tests showed prolonged prothrombin time and PT-INR 2.63 before 4F-PCC administration (test point A). 4F-PCC induced a rapid reduction of PT-INR to 1.22 at one hour after administration (test point B). PT-INR was 1.69 after administration of protamine following withdrawal from cardiopulmonary bypass (test point C). Platelets (20 U) and FFP (8 U) were transfused, and fibrinogen (3 g) was administered (test point D). Platelets (20 U) and FFP (8 U) were transfused, and fibrinogen (2 g) was administered additionally (test point E). PT-INR: prothrombin time-international normalized ratio; Vit. K: vitamin K; CPB: cardiopulmonary bypass; Plt: platelet; Fib: fibrinogen; FFP: fresh frozen plasma; U: units; ACT: activated clotting time; TEG: thromboelastogram

**Figure 2:**
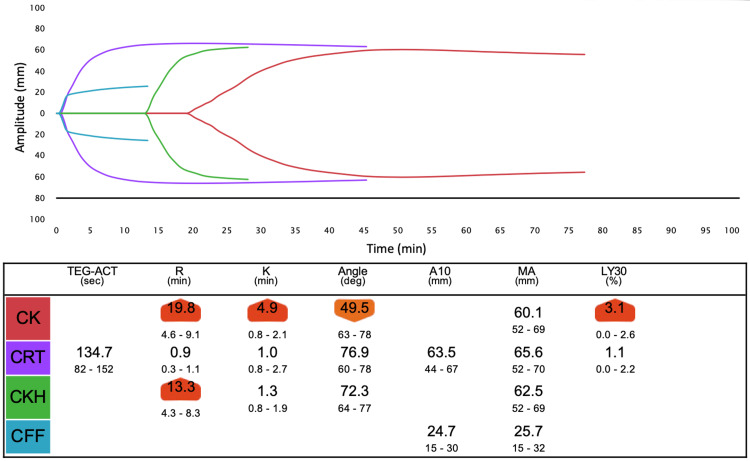
Overview of TEG6s data before 4F-PCC administration at test point A. The citrated kaolin (CK) assay containing the coagulation activator, kaolin, which mainly activates the intrinsic pathway, identifies hemostatic characteristics. The citrated rapid thromboelastogram (CRT) assay, containing tissue factor and kaolin, assesses hemostasis properties more rapidly. The citrated kaolin with heparinase (CKH) assay, containing kaolin and heparin-neutralizing enzyme, assesses hemostatic characteristics without the influence of heparin. Citrated functional fibrinogen (CFF) assay isolates fibrinogen function, which contributes to clot strength. R (minutes) is the time until thrombus amplitude reaches 2 mm. K (minutes) is the time from the thrombus amplitude reaching 2 mm until the amplitude reaches 20 mm. The angle is the slope of a tangent line from the tracing at the midpoint between the R and K times. A10 is the thrombus amplitude at 10 minutes, and MA is the absolute thrombus strength. LY30(%) represents the percentage of fibrinolysis at 30 minutes after max amplitude.

**Figure 3:**
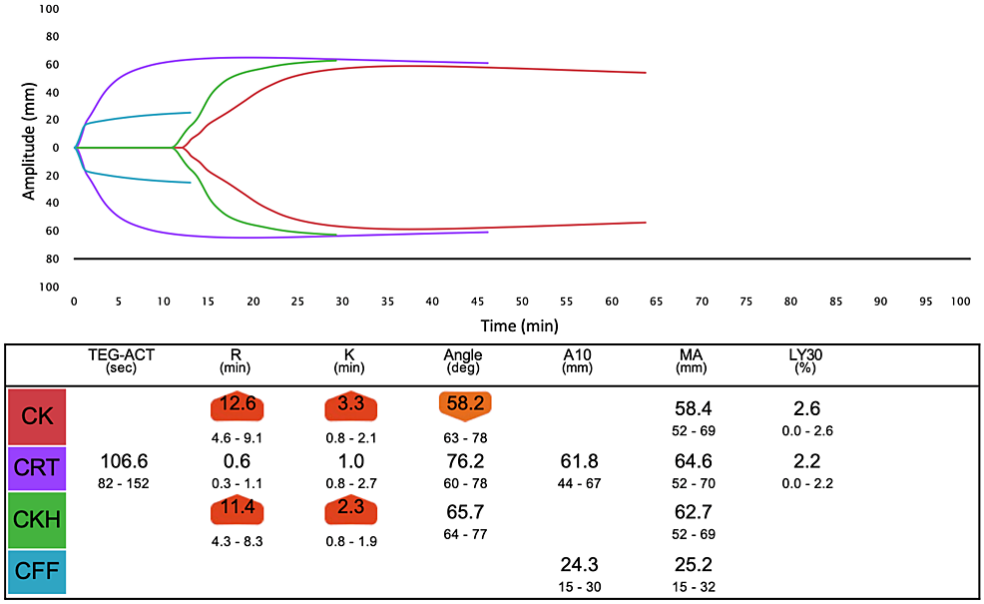
Overview of TEG6s data one hour after 4F-PCC administration (test point B).

**Figure 4:**
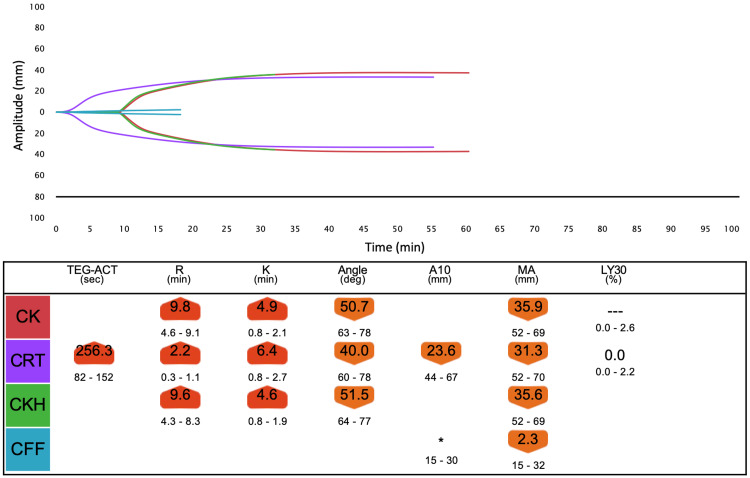
Overview of TEG6s data after protamine administration following withdrawal from cardiopulmonary bypass (test point C).

**Figure 5:**
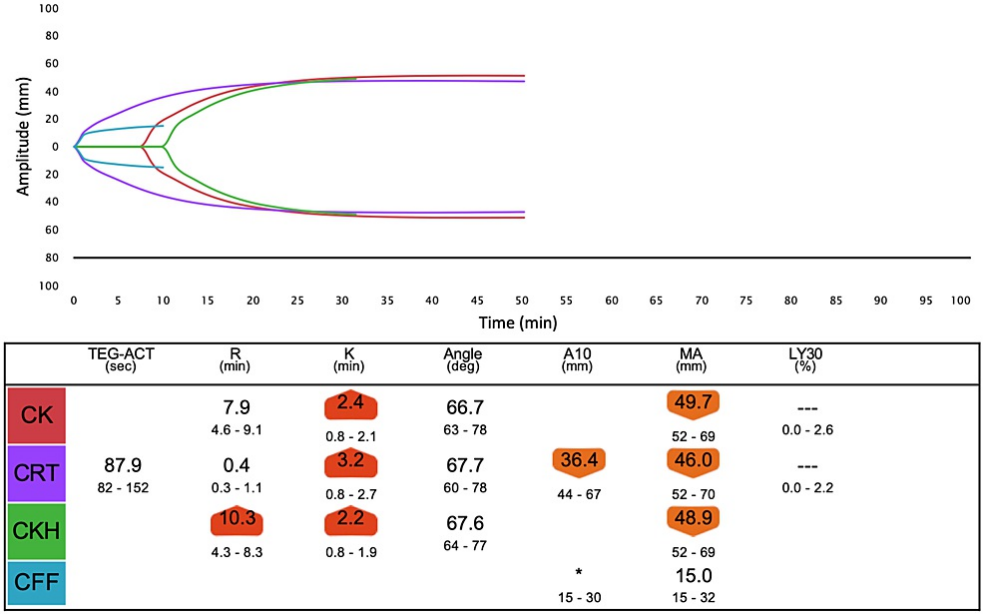
Overview of TEG6s data after platelet (20 U) and FFP (8 U) transfusion, and fibrinogen (3 g) administration (test point D). FFP: fresh frozen plasma

**Figure 6:**
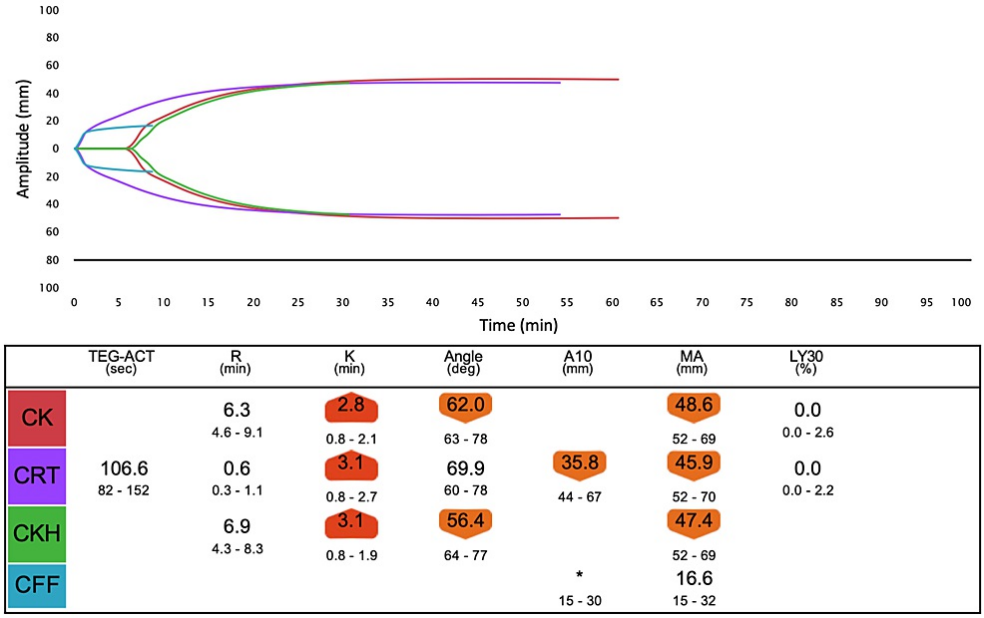
Overview of TEG6s data after additional platelet (20 U) and FFP (8 U) transfusion, and fibrinogen (2g) administration (test point E). FFP: fresh frozen plasma

We assessed the hemostatic status using TEG6s (Haemonetics Corporation, USA) during surgery. Test points of the TEG6s test, time change of PT-INR, and other related tests are shown in Figure 1. From the value of TEG6s before 4F-PCC administration, coagulation function was inhibited probably due to warfarin and heparin (Figure 2). R time in TEG6s improved one hour after 4F-PCC administration (Figure 3). R time was slightly improved after protamine administration compared to the values before cardiopulmonary bypass (Figure 4). Blood tests showed a similar tendency. Fresh frozen plasma (FFP), platelets, and 3 g of fibrinogen were administered because of the reduction of platelet and fibrinogen. Then, the R time and max amplitude in each assay were preserved near the lower limit of the normal range (Figures 5, 6).

The anesthesia time was 604 minutes, the operation time was 522 minutes, and the cardiopulmonary bypass time was 183 minutes. The total calculated blood loss was 2,850 mL. Anticoagulation therapy with warfarin was resumed on postoperative day three. There were only minor hemorrhagic complications without symptoms, and no obvious thrombotic events occurred. The patient completed the rehabilitation program and was discharged three months after the surgery.

## Discussion

This is the first case report that describes the observation of the effects of 4F-PCC by TEG6s in a patient who underwent LVAD conversion surgery. In a previous retrospective study of 4F-PCC in patients with LVAD who underwent invasive procedures except for LVAD conversion (n = 49), 4F-PCC significantly decreased the mean INR and no thrombotic events were observed [6]. Warfarin reversal with 4F-PCC was reported to be superior or non-inferior to FFP in hemostasis and risk of thromboembolism [1,2,7,8]. It had a lower risk of volume overload compared to FFP. Furthermore, it does not require ABO typing and does not cause allosensitization, which is a concern for patients bridging to cardiac transplantation. Furthermore, 4F-PCC could be more cost-effective than FFP [9]. Single vitamin K administration requires time to obtain the effects and can result in a sustained, delayed, or unpredictable reversal of anticoagulation. Bridging with unfractionated heparin has little evidence to prevent pump thrombus. Therefore, warfarin reversal of 4F-PCC can be feasible, effective, and safe for LVAD patients. In our case, 4F-PCC with co-administration of vitamin K achieved good hemostasis and lower blood loss than expected. Concomitant use of vitamin K is recommended to sustain the effects of 4F-PCC because of the relatively short half-life of the latter [10]. However, there are no reports comparing the effects of vitamin K with and without concomitant use with 4f-PCC.

Careful monitoring of coagulation is necessary to prevent overcoagulation. POC monitoring has faster turnaround times and a better ability to identify specific coagulation defects, thereby allowing for targeted and more rapid management of coagulopathic bleeding compared to standard hemostatic tests [11]. We measured TEG6s as a POC monitor in addition to PT-INR and APTT. We expected the R time in TEG6s to be most affected by 4F-PCC and warfarin, similar to PT-INR. R time is the time from the start of analysis until the thrombus amplitude reaches 2 mm, which corresponds to the onset of coagulation caused by coagulation factors. In fact, R time was clearly prolonged before 4F-PCC administration (Figure 2) and improved after 4F-PCC administration, similar to PT-INR (Figure 3). However, after protamine administration, although R time was further reduced, PT-INR was mildly prolonged (Figure 4). Therefore, there may be a limitation in that the results from TEG6s are also affected by other coagulation factors not related to warfarin. However, multiple factors, including fibrinogen and platelet, can affect the coagulation system in LVAD surgery, as in this case. TEG has other parameters to assess them and the great advantages of establishing a treatment strategy.

## Conclusions

4F-PCC and co-administration of vitamin K provided good hemostasis in our case. Although the indication of 4F-PCC for warfarin reversal is only in urgent surgery, 4F-PCC can be a useful method for elective surgery, as in this case, which has both risks of massive bleeding and thrombosis, and it is necessary to minimize the time of warfarin reversal. TEG6s can be useful for frequent monitoring of the coagulation status to assess the efficacy and complications of 4F-PCC, although further clinical research is necessary.
